# Perylene bisimide hydrogels and lyotropic liquid crystals with temperature-responsive color change†
†Electronic supplementary information (ESI) available: Detailed procedures and results for all reported experiments, along with synthetic details for **PBI 1**. See DOI: 10.1039/c6sc02249a



**DOI:** 10.1039/c6sc02249a

**Published:** 2016-07-22

**Authors:** Daniel Görl, Bartolome Soberats, Stefanie Herbst, Vladimir Stepanenko, Frank Würthner

**Affiliations:** a Institut für Organische Chemie & Center for Nanosystems Chemistry , Universität Würzburg , Am Hubland , 97074 Würzburg , Germany . Email: wuerthner@chemie.uni-wuerzburg.de

## Abstract

The self-assembly of perylene bisimide (PBI) dyes bearing oligo ethylene glycol (OEG) units in water affords responsive functional nanostructures characterized by their lower critical solution temperature (LCST). Tuning of the LCST is realized by a supramolecular approach that relies on two structurally closely related PBI–OEG molecules. The two PBIs socially co-assemble in water and the resulting nanostructures exhibit a single LCST in between the transition temperatures of the aggregates formed by single components. This permits to precisely tune the transition from a hydrogel to a lyotropic liquid crystal state at temperatures between 26 and 51 °C by adjusting the molar fraction of the two PBIs. Owing to concomitant changes in PBI–PBI interactions this phase transition affords a pronounced color change with “fluorescence-on” response that can be utilized as a smart temperature sensory system.

## Abstract 2

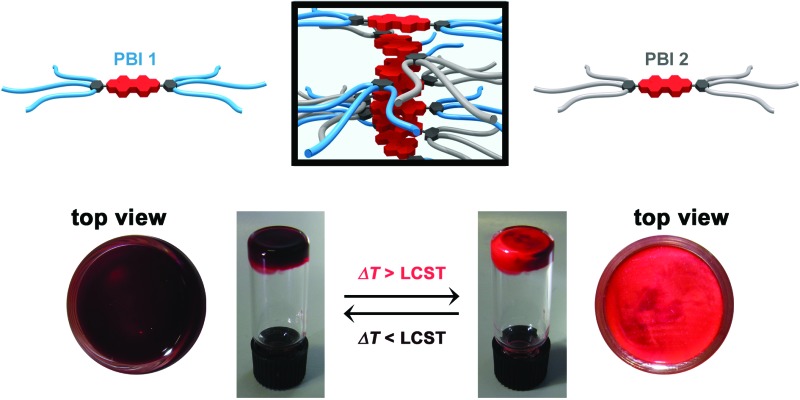
Tuning of the temperature response of perylene bisimide hydrogels exhibiting LCST behavior has been achieved by social self-assembly in water.

## Introduction

Molecular self-assembly has attracted a great deal of attention as an elegant and inexpensive approach for fabricating nanostructured functional materials.^1^ In particular, self-assembly of π-conjugated dye molecules has intensively been studied,^2^ because π–π-interactions between dyes lead to peculiar changes of electronic and optical properties which holds great promise for applications in organic electronics,^3^ photonics^4^ and sensing.^5^ Materials composed of dye aggregates have recently also gained much interest in an aqueous environment because the intermolecular interactions between the π-conjugated chromophores are remarkably enhanced due to the hydrophobic effect.^6^ The majority of the so far investigated π-amphiphilic molecules rely on oligoethylene glycol (OEG) as a water solubility providing element. Interestingly, this structural motif can provide additional features to the material such as formation of hydrogels at higher concentration and lower critical solution temperature (LCST) behaviour.^7^ LCST is a phenomenon based on the entropy-driven phase transition of an at least binary mixture at a specific temperature due to changes in the noncovalent interactions with surrounding solvent molecules. However, the relevance of LCST behaviour for self-assembled discrete molecules has only recently come into focus.^8–10^ It is of special interest in π-conjugated systems because the structural changes accompanying the phase transition can be used to trigger an optical or electronic response, as has been shown for oligo(*p*-phenylene) derivatives^9^ and photo-switchable diarylethenes.^10^ In this context, the use of perylene bisimide (PBI) dyes is very attractive due to their outstanding optical and self-assembly properties.^2f,11^ In our recent work on **PBI 2** self-assembly in water we noted an unexpected high solubility at lower temperatures,^12*a*^ but precipitation at higher temperatures, *i.e.* LCST behaviour.^12*b*^ Our subsequent research was accordingly devoted to reveal the origin of this effect which led to several interesting discoveries in the field of amphiphilic dyes.

**Fig. 1:**
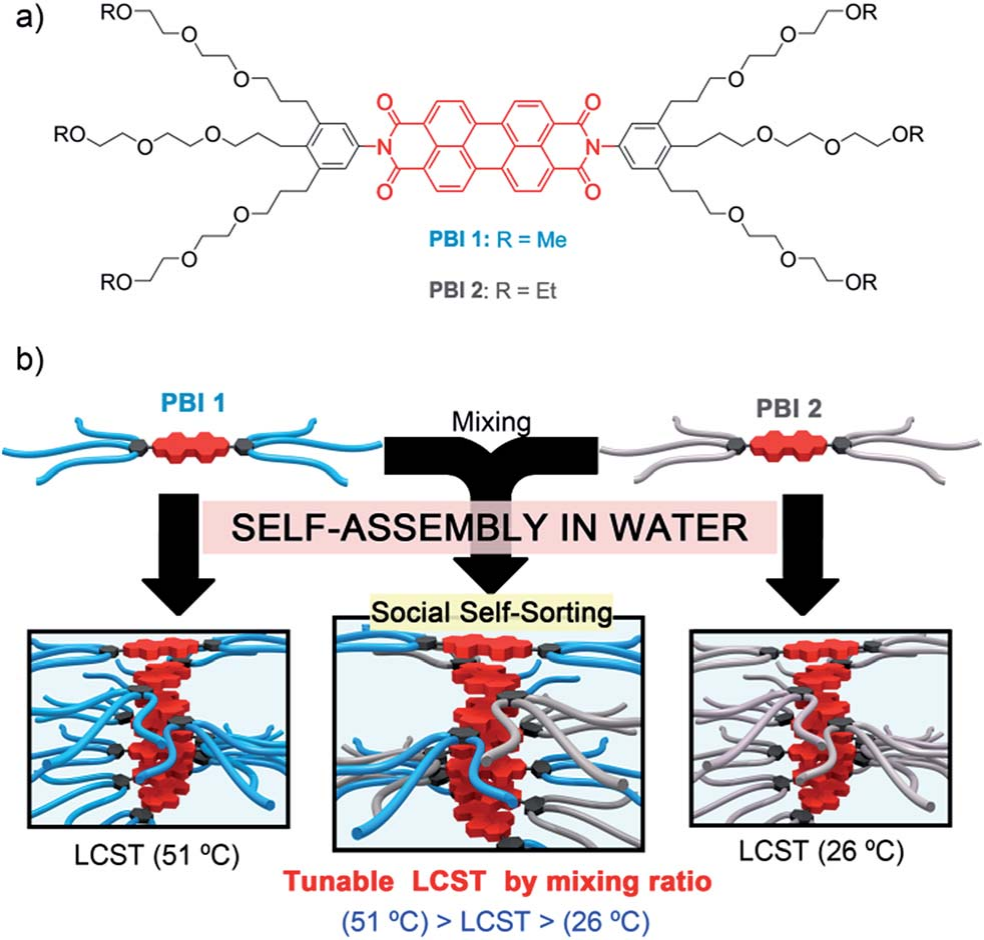
(a) Chemical structures of **PBI 1** and **PBI 2**. (b) Schematic illustration of the self-assembly features and LCST properties of **PBI 1**, **PBI 2** and **PBI 1**/**PBI 2** co-aggregates.

Herein we report on hydrogel and lyotropic liquid crystal mesophase formation and a novel supramolecular approach to tune the LCST properties in self-assembled aggregates of bolaamphiphilic PBI dyes. Fluorescent **PBI 1** and **PBI 2** differ only in a methylene bridge in the OEG termini (Fig. 1a) but their aqueous aggregates exhibit remarkably different LCSTs (Fig. 1b). This unique feature allows the creation of **PBI 1**/**PBI 2** co-aggregates with a single LCST phase transition that is precisely tuned between 26 and 51 °C by adjusting the molar fraction of the two PBI components (Fig. 1b). This unprecedented strategy is further applied for the development of tunable temperature responsive **PBI 1**/**PBI 2** hydrogels that afford a remarkable color change during LCST phase transition, overall highlighting the impact of implementing small structural changes at the molecular periphery for fine-tuning of the LCST.

## Results and discussion

In the present work, we synthesized the new PBI–OEG derivative **PBI 1** that differs from the previously reported **PBI 2** (ref. 12) by one methylene unit at the termini of the OEG side chains (Fig. 1a) (for synthetic details, see the ESI†). According to their structural similarity, **PBI 1** and **PBI 2** exhibit identical optical and aggregation properties, but surprisingly show a strikingly distinct LCST behaviour in water, which has been of great relevance for the LCST tuning in the mixtures described below.

We have previously shown that **PBI 2** self-organizes hierarchically into columnar and lamellar superstructures in water, which are soluble at room temperature but precipitate at increased temperatures, indicative of LCST behaviour.^12^ For **PBI 1**, we found a similar aggregation behaviour like for **PBI 2** (Fig. S1†). For example, the UV-vis absorption spectra of both compounds in THF show the vibronic fine structure commonly observed for monomerically dissolved PBI dyes, whereas the UV-vis spectra in water reveal a hypsochromically shifted absorption maximum concomitant with a loss of the vibronic fine structure, indicating the self-assembly of the PBIs into well-defined H-aggregates. Moreover, both compounds exhibit almost identical extinction coefficients and fluorescence quantum yields in both THF (*Φ*_fl_(**PBI 1**) = 42%, *Φ*_fl_(**PBI 2**) = 43%) and in pure water (Fig. S1†). These results confirm that **PBI 1** and **PBI 2** self-assemble in water with the same chromophore arrangement, forming long aggregates even at nanomolar concentrations.^12^

**Fig. 2:**
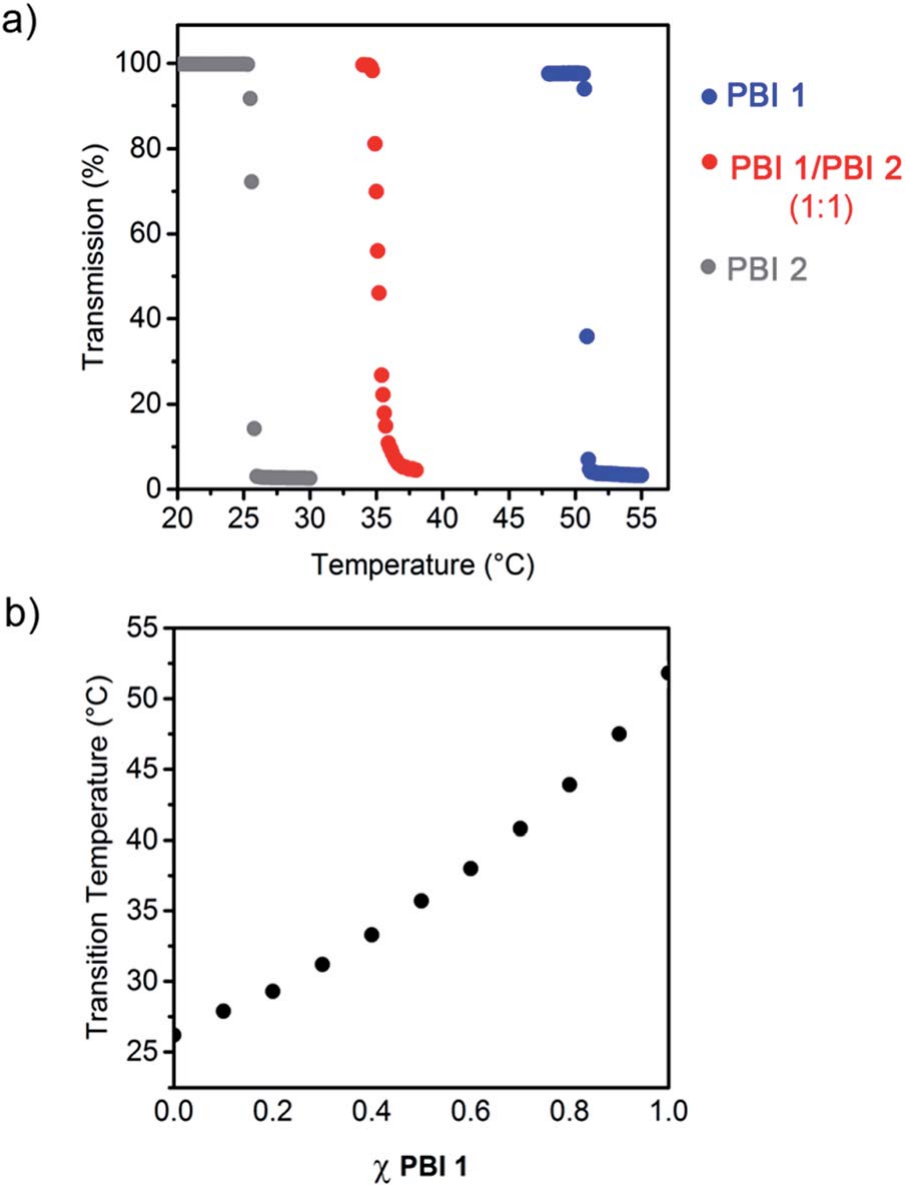
(a) Transmission at 800 nm of aqueous solutions (2.5 × 10^–4^ M) of **PBI 1**, **PBI 2** and **PBI 1**/**PBI 2** (1 : 1) as a function of the temperature. Heating rate 0.1 °C min^–1^. (b) Phase transition temperatures for **PBI 1**/**PBI 2** aggregates (2.5 × 10^–4^ M) as function of the molar fraction (*χ*) of **PBI 1**.

The temperature response of **PBI 1** and **PBI 2** aggregates was examined by turbidity measurements in water (*c* = 2.5 × 10^–4^ M) (Fig. 2a and S2†). These experiments revealed abrupt drops in the transmission at 800 nm after exceeding a certain temperature (Fig. 2a). This behaviour is attributed to the temperature dependent dehydration of the OEG chains which provoke the phase separation of the aggregates and water, which is known as critical solution temperature phenomenon.^7^ In the present work, the onset temperature of this phase transition (Fig. S2†) will be referred as LCST. Interestingly, it was found that **PBI 1** exhibits a phase transition at 51 °C, while we previously reported that **PBI 2** exhibits a LCST transition at 26 °C (Fig. 2a and S2†). Hence, small differences in the molecular structure such as the replacement of ethyl by methyl end groups (Fig. 1a) have remarkable impact on the LCST behaviour, which has barely been addressed in literature so far.^13^ The heating curves are highly reproducible, which suggests that both dyes form thermodynamically stable aggregates in water.

Inspired by previous studies on random copolymers, which showed that the LCST behavior can be tuned by the control of the ratio of two different polymer building blocks,^7^ we hypothesized that **PBI 1** and **PBI 2** would socially self-assemble to form co-aggregated structures^14^ exhibiting a LCST behaviour dependent of the PBIs mixing ratio. Accordingly, different aqueous solutions of **PBI 1**/**PBI 2** mixtures were prepared and their transmission at 800 nm was monitored as a function of temperature (Fig. 2 and S2†). For example, the 1 : 1 solution of **PBI 1** and **PBI 2** exhibits a single transition at 35 °C (Fig. 2a), which is a strong indication that both dyes perfectly mix with each other even without an orthogonal binding motif, thus forming socially self-sorted heteroaggregates.^14^ Pleasingly, the LCSTs of the **PBI 1**/**PBI 2** system is consequently adjusted in between the temperature interval ranging from 26 °C (LCST of **PBI 2** aggregate) to 51 °C (LCST of **PBI 1** aggregate) depending on the molar fraction of both dyes (Fig. 2b). Such control of the LCST properties in co-aggregated structures of discrete molecules is indeed unprecedented but constitutes a facile and efficient strategy to fine-tune temperature responsive systems. Therefore the LCST seems to be a powerful tool for the investigation of supramolecular co-assemblies.

**Fig. 3:**
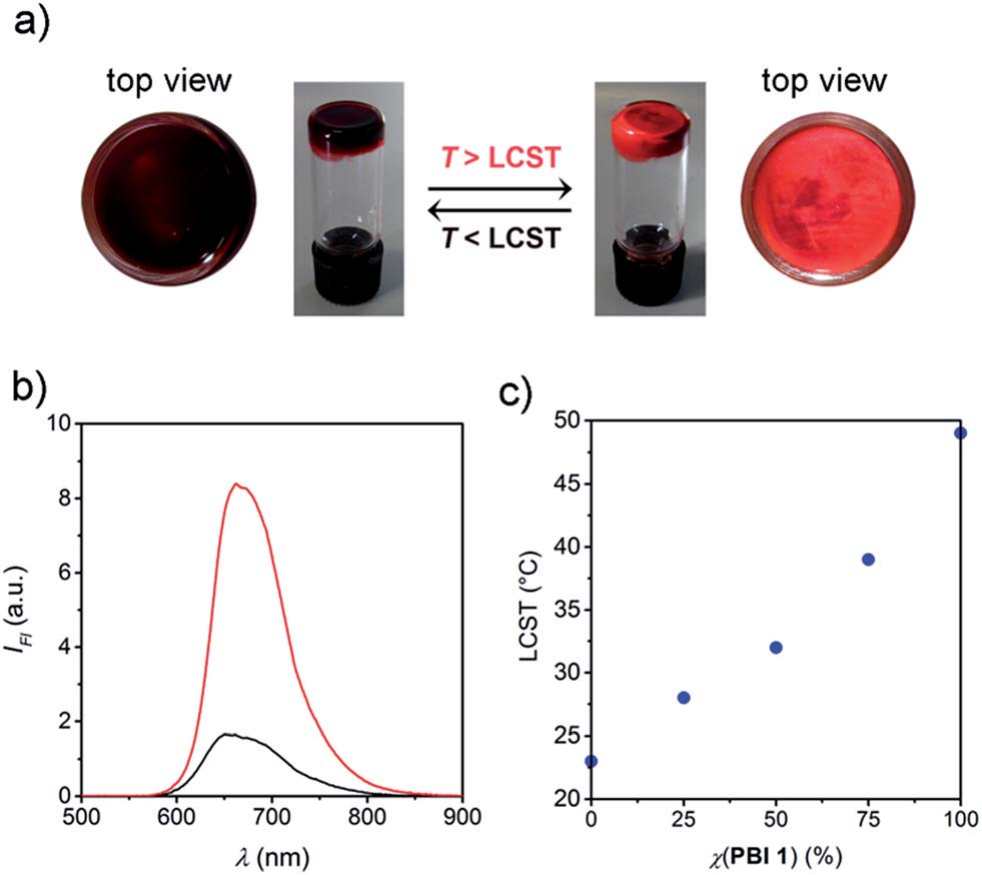
(a) Photographs illustrating the color change of **PBI 2** hydrogel (80 wt% water content) upon phase transition (*ca*. 26 °C). (b) Fluorescence spectra of **PBI 1** in water (80 wt%) before (21 °C, blue) and after (56 °C, red) LCST phase transition. *λ*_ex_ = 380–420 nm. (c) Phase transition temperatures of **PBI 1**/**PBI 2** hydrogels (80 wt% water content) as function of the molar fraction (*χ*) of **PBI 1**.

In the aim to demonstrate that this strategy can be applied for the development of stimuli responsive materials, we focused on highly concentrated aqueous materials based on **PBI 1** and **PBI 2**. Both PBIs exhibit thermotropic columnar liquid crystalline phases under anhydrous conditions (Fig. S3 and S4†), whereas they form lyotropic columnar hexagonal phases upon addition of 20 wt% of water (Fig. S5 and S6†).^15^ After further addition of water up to 80 wt% water content, the dye molecules form hydrogels at room temperature. Most interestingly, these hydrogels are subject to LCST behaviour at temperatures of 51 (for **PBI 1**) and 26 °C (for **PBI 2**) which are accompanied by a significant color change from dark red to bright orange-red that can easily be followed by the naked eye (Fig. 3a). It is noteworthy that this process is reversible and proceeds in quasisolid state without any appreciable volume change.

In order to unravel the origin of the color change we next conducted temperature-dependent fluorescence measurements of hydrogel films by fluorescence microscopy (ESI†). Indeed, the fluorescence emitted at the surface of the hydrogels is enhanced remarkably upon LCST phase transition (Fig. 3b and S7†) and resembles the excimer-type emission known for **PBI 2** aggregates,^12^ which is consistent with the formation of face-to-face stacked PBI assemblies (Fig. 1b). The fluorescence intensity is increased by a factor of ∼7, which we attribute to a rigidification effect,^16^ *i.e.* decrease in the rotational and translational motion of the stacked PBI molecules. These motions, which are known as fluorescence quenching pathways in photoexcited PBI aggregates,^17^ are less restricted below the LCST, in which the OEG chains are solvated. Also for less concentrated samples (*c* = 8 × 10^–5^ M) an increase in fluorescence intensity has been observed upon LCST phase transition (Fig. S8a and c†). Corresponding absorption measurements revealed insignificant changes of the absorption profile, while scattering effects become pronounced due to the turbidity of the sample (Fig. S8b and d†). Accordingly, the thermally induced color change in PBI hydrogels is mainly attributed to an increase of the excimer-type fluorescence intensity (Fig. 3b, S7 and S8†) as well as other factors such as changes in light scattering and diffraction indices induced by the phase separation (*vide infra*).

In order to prove the tunability of the LCSTs of **PBI 1**/**PBI 2** mixtures in the hydrogel state, we prepared mixtures of 3 : 1, 1 : 1 and 1 : 3 ratios of **PBI 1**/**PBI 2** and we examined their temperature behaviour by differential scanning calorimetry (DSC) measurements (Fig. S9†). These experiments revealed indeed only one phase transition for each mixture, which is accompanied by a single, fast and reversible color change that can be easily followed by naked eye. Like in case of the diluted samples, the LCST phase transition in the **PBI 1**/**PBI 2** hydrogels occurs at a temperature in between 26 (LCST of **PBI 2**) and 51 °C (LCST of **PBI 1**) (Fig. 3c). Accordingly, the LCST process in **PBI 1** and **PBI 2** is not influenced by the concentration but highly dependent on the mixing ratio of the PBIs. It is noteworthy that the optical response is identical for **PBI 1**, **PBI 2** and for their corresponding mixtures, because the two PBIs differ in the OEG-chains which does not influence the electronic coupling between the photoactive part of the self-assembled structure (PBI-cores).^18^ Therefore, the small structural changes in the molecular periphery allow to affect LCST transition temperatures while keeping the optoelectronic and self-assembly properties of the PBI material unchanged.

**Fig. 4:**
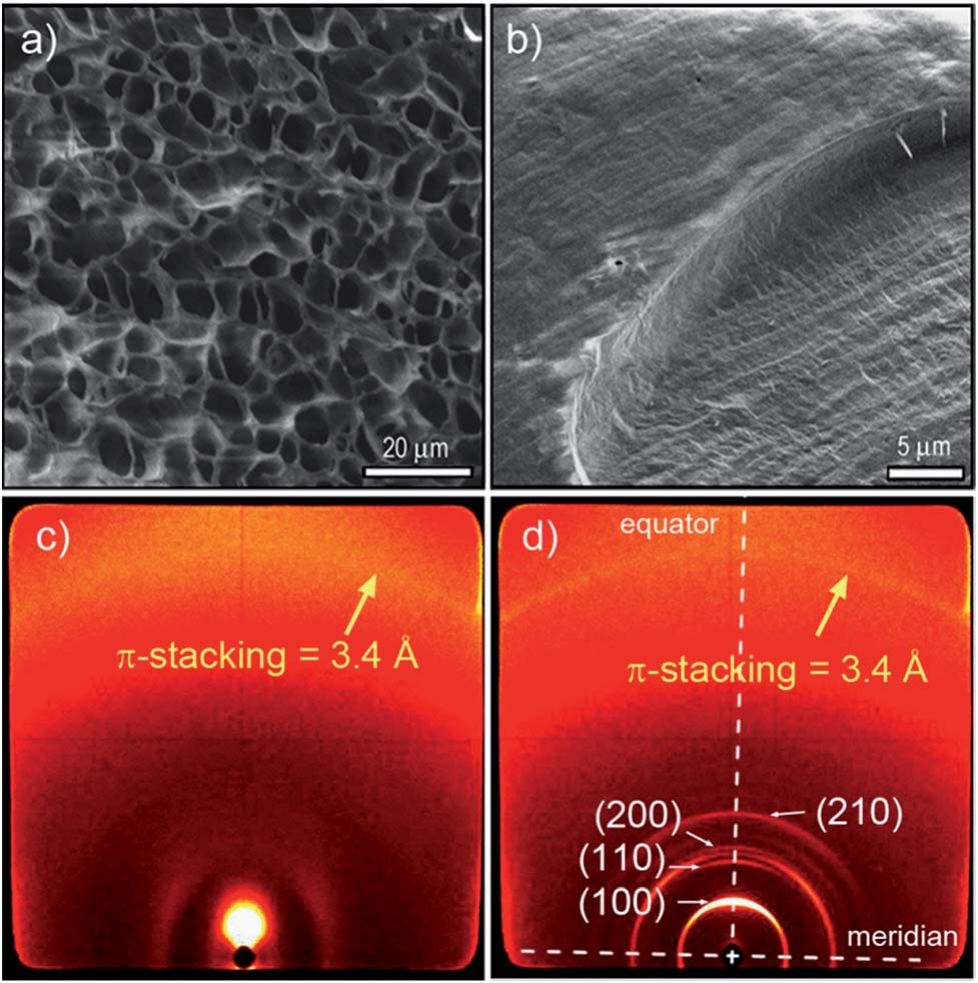
Cryo-SEM images of **PBI 1** in H_2_O (80 wt% water content) (a) below LCST and (b) above LCST. Corresponding 2D X-ray diffraction patterns of **PBI 1** in H_2_O (80 wt% water content) obtained at (c) 35 °C (below LCST) and (d) 70 °C (above LCST), respectively.

**Fig. 5:**
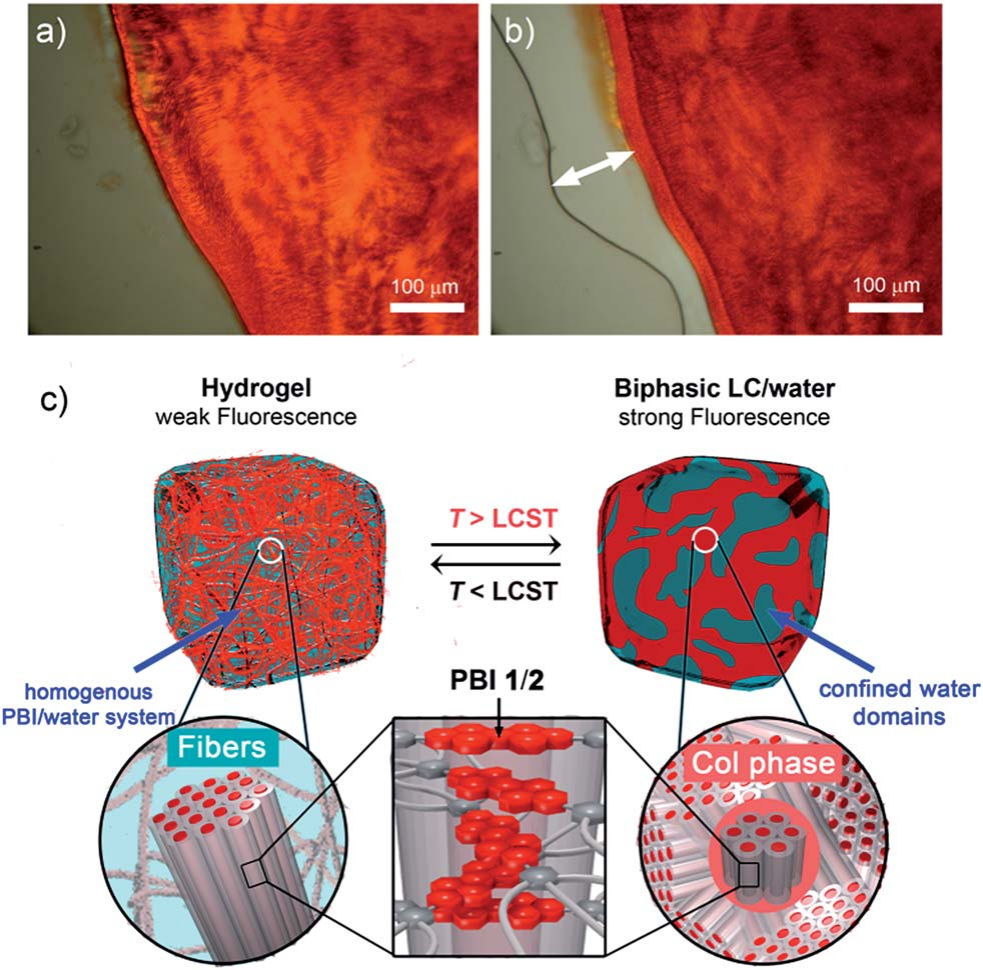
Optical microscope images of **PBI 1** in H_2_O (80 wt% water content) (a) below LCST (40 °C) and (b) above LCST (58 °C). The release of water at the periphery of the gel is indicated with a white arrow. (c) Schematic illustration of the LCST induced phase transition in **PBI 1** and **PBI 2** hydrogels. Col: columnar nanostructure.

To gain insight into the origin of this LCST phase transition in these hydrogels, we have examined the structural features of **PBI 1** and **PBI 2** with 80 wt% water content below and above the LCST by means of scanning electron microscopy (SEM), X-ray diffraction (XRD) and optical microscopy (OM) (Fig. 4, 5 and S10–S15†). Fig. 4a and b show the cryo-SEM images of **PBI 1** hydrogel below and above LCST, respectively, which reveal a significant morphology change upon LCST phase transition. A porous network with rather random branching has been obtained below LCST (Fig. 4a, S10a, b and S11a, b†), which is common for gel materials.^19^ In contrast, above the critical temperature a compact and non-porous structure consisting of bundled aggregate strands with a diameter of up to 400 nm (Fig. 4b, S10c, d and S11c, d†) is observed. These results indicate that **PBI 1** forms a higher ordered state after LCST phase transition.

This observation was further corroborated by X-ray scattering below LCST and above LCST (Fig. 4c and d and S12†). The 2D X-ray patterns of **PBI 1** with 80 wt% water content at 35 °C (below LCST) show no reflection in the small angle region (Fig. 4c), which indicates the absence of a well-ordered nanostructure. In contrast, the X-ray pattern of the same sample at 70 °C exhibits one intense and three weak reflections along the equator corresponding to the (100), (110), (200) and (210) lattices of a columnar hexagonal phase (Fig. 4d).

Corresponding OM images of **PBI 1** hydrogel revealed a homogenous phase below the LCST (Fig. 5a), but after heating above the LCST a biphasic mixture consisting of a red phase and a transparent liquid (water) is observed (Fig. 5b). These microscope observations show that the phase transition is accompanied by the expulsion of water from the gel matrix, which likely triggers the temperature-induced transition between the hydrogel state and a biphasic state consisting of pure water and a nanostructured columnar PBI phase (Fig. 5c).

This LCST transition behaviour was found in hydrogels of **PBI 1**, **PBI 2** as well as in **PBI 1**/**PBI 2** mixtures (Fig. S13–S15†) and is always accompanied by the remarkable color change. It should be noted that the biphasic state observed above LCST exhibits a similar X-ray pattern to that observed for the lyotropic columnar hexagonal phase (20 wt% water content) of the same compound (Fig. S5†). For the latter, temperature-induced water expulsion has not been observed. Accordingly, a rather high concentration of water is required for the formation of **PBI 1** and **PBI 2** self-assemblies that are subject to LCST phase transition.

## Conclusion

In summary, we have developed a new supramolecular strategy to tune LCST properties in self-assembled aggregates of OEG-substituted dyes. **PBI 1** and **PBI 2** differ only in one methylene group at the peripheral ends of the OEG side chains, but their aggregated structures in water exhibit remarkably different LCST properties. This unique feature allows for the development of mixed hydrogel materials with only one phase transition due to the social co-assembly of **PBI 1** and **PBI 2** into supramolecular co-aggregates. The phase transition of these hydrogels is accompanied by a remarkable color change from dark red to bright orange-red that can accordingly be adjusted to any temperature between 51 and 26 °C by controlling the molar ratio of **PBI 1** and **PBI 2**. Taking into account that the LCSTs of the present hydrogels cover environmentally and physiologically relevant temperatures, we foresee smart sensory systems based on these novel stimuli responsive materials. Applications in (bio-)medical sensing^20^ and health monitoring^21^ should be possible as well if our concept can be extended to other self-assemblies of π-conjugated molecules.

## References

[cit1] Lehn J.-M. (2013). Angew. Chem., Int. Ed..

[cit2] Kim H. J., Kim T., Lee M. (2011). Acc. Chem. Res..

[cit3] Jain A., George S. J. (2015). Mater. Today.

[cit4] Li H. G., Choi J., Nakanishi T. (2013). Langmuir.

[cit5] Thomas S. W., Joly G. D., Swager T. M. (2007). Chem. Rev..

[cit6] Molla M. R., Ghosh S. (2014). Phys. Chem. Chem. Phys..

[cit7] Li C., Liu S. (2012). Chem. Commun..

[cit8] Wei P., Cook T. R., Yan X., Huang F., Stang P. J. (2014). J. Am. Chem. Soc..

[cit9] Kim Y., Li W., Shin S., Lee M. (2013). Acc. Chem. Res..

[cit10] Hirose T., Matsuda K., Irie M. (2006). J. Org. Chem..

[cit11] Würthner F., Saha-Möller C. R., Fimmel B., Ogi S., Leowanawat P., Schmidt D. (2016). Chem. Rev..

[cit12] Zhang X., Görl D., Stepanenko V., Würthner F. (2014). Angew. Chem., Int. Ed..

[cit13] Ishizone T., Seki A., Hagiwara M., Han S. (2008). Macromolecules.

[cit14] Safont-Sempere M. M., Fernández G., Würthner F. (2011). Chem. Rev..

[cit15] Rodler F., Schade B., Jäger C. M., Backes S., Hampel F., Böttcher C., Clark T., Hirsch A. (2015). J. Am. Chem. Soc..

[cit16] An B.-K., Kwon S.-K., Jung S.-D., Park S. Y. (2002). J. Am. Chem. Soc..

[cit17] Sung J., Kim P., Fimmel B., Würthner F., Kim D. (2015). Nat. Commun..

[cit18] Würthner F. (2004). Chem. Commun..

[cit19] Babu S. S., Prasanthkumar S., Ajayaghosh A. (2012). Angew. Chem., Int. Ed..

[cit20] Sun M., Müllen K., Yin M. (2016). Chem. Soc. Rev..

[cit21] Chortos A., Bao Z. (2014). Mater. Today.

