# Supplementary material for: Perylene bisimide hydrogels and lyotropic liquid crystals with temperature-responsive color change
Source: Chem Sci. 2016 Jul 22;7(11):6786–90. doi: 10.1039/c6sc02249a (PMC5356028; doi:10.1039/c6sc02249a)
Supplement: Supplementary file 1 [file SC-007-C6SC02249A-s001.pdf]

Electronic Supplementary Material (ESI) for Chemical Science.
This journal is © The Royal Society of Chemistry 2016

Electronic Supplementary Information

Perylene bisimide hydrogels and lyotropic liquid crystals with temperature-responsive color change

Daniel Görl, Bartolome Soberats, Stefanie Herbst, Vladimir Stepanenko and Frank Würthner*

Institut für Organische Chemie & Center for Nanosystems Chemistry

Universität Würzburg

Am Hubland, 97074 Würzburg (Germany)

E-mail: wuerthner@chemie.uni-wuerzburg.de

1. Materials and Methods

NMR spectroscopy. ^{1}H and ^{13}C nuclear magnetic resonance (NMR) spectra were recorded on a Bruker AV 400 spectrometer using $CDCl_3$ as solvent. The chemical shifts are reported in ppm and refer to the proton signal of the solvent as internal standard. Multiplicities for proton signals are abbreviated as s, d, and m for singlet, doublet, and multiplet, respectively.

Mass spectrometry. High-resolution mass spectra (HRMS) were recorded on a micrOTOF focus (Bruker Daltonik GmbH, Germany).

UV/Vis spectroscopy. UV/Vis absorption spectra were recorded using a Perkin Elmer Lambda 40P spectrophotometer. The spectra were measured in quartz glass cuvettes using either spectroscopic grade tetrahydrofuran (Uvasol®) or deionized water prepared by water purification system PURELAB classic (ELGA, France). Temperature control was accomplished by Perkin Elmer PTP-1+1 Peltier system. Extinction coefficients were calculated from Lambert-Beer's law.

Fluorescence spectroscopy. Fluorescence spectra were recorded on a PTI QM-4/2003 fluorescence spectrometer and are corrected against photomultiplier and lamp intensity. Fluorescence quantum yield (Φ) of **PBI 1** was calculated from the integrated intensity under the emission band (I) using the following equation:

$$\Phi = \Phi_r \frac{I \cdot OD_r \cdot n^2}{I_r \cdot OD \cdot n_r^2}$$

Where OD is the optical density of the solution at the excitation wavelength and n is the refractive index. The optical density of the solution for the calculation of quantum yields was less than 0.05 at the excitation wavelength. *N*,*N*'-Bis(2,6-diisopropylphenyl)-1,6,7,12-tetraphenoxyperylene-3,4:9,10-tetracarboxylic acid bisimide (Φ_r = 0.96) in chloroform was used as reference.[S1]

Fluorescence spectra of hydrogel samples were recorded with an ECLIPSE LV100 POL polarizing microscope (Nikon, Japan) using scope mode, ten-fold magnification and two seconds exposure time. An Intenslight C-HGFI mercury vapor lamp (Nikon, Japan) served as external light source. The spectra are averaged from ten measurements. Excitation wavelength region was 380-420 nm. The hydrogel sample was carefully placed as a film between two glass plates.

Cryogenic scanning electron microscopy. Cryo-SEM measurements were performed using a Zeiss Ultra Plus Field Emission SEM operating at 2-5 kV with an aperture size set of 30 μm to avoid excessive charging and radiation damage of imaged areas. Sample preparation consisted of placing a small drop of the PBI hydrogel onto copper stub sample holder. Prior to examination, the specimen was plunged into liquid nitrogen slush at -210 °C. The sample was then transferred under vacuum using the loading transfer rod into the high vacuum cryo-preparation chamber (Quorum PP2000T) at -180 °C, fractured and then transferred into a SEM sample chamber maintained at about -150 °C.

Polarizing optical microscopy (POM) and optical microscopy (OM). The materials were examined under an Olympus Bx41 (Nikon, Japan) polarizing microscope equipped with a THMS 600 heating stage (Linkam, Great Britain).

Wide-angle X-ray scattering. Temperature-dependent WAXS measurements were performed on a Bruker Nanostar (Detector Vantec2000, Microfocus copper anode X-ray tube Incoatec). Thermotropic and lyotropic liquid-crystalline samples were prepared by fiber extrusion using a mini-extruder. The measurements were carried out in Mark capillaries (Hilgenberg) positioned perpendicular to the incident X-ray beam.

Differential Scanning Calorimetry (DSC). DSC spectra were recorded with a DSC Q1000 (TA Instruments, Germany).

The hydrogel samples for the XRD measurements were prepared as follows: The viscous PBI hydrogels (80 wt% water content) were placed inside a glass Mark-tube (Ø=1.5 mm). The tube, open at both sides, was placed vertically in a vial to allow the sample to reach the middle part of the tube by gravity (≈6 h). Then, the tube was sealed by melting both sides. This tube was inserted into another Mark-tube (Ø=2 mm) which was also sealed by melting. This sample preparation prevented the evaporation of the water during the measurements that were performed under vacuum.

It should be noted that a certain degree of alignment is observed in the 2D X-ray diffraction patterns of PBI hydrogels (80 wt% water content) above the LCST (Figure 4e and Figure S10b). The reflections corresponding to the columnar hexagonal lattices appear along the equator, which indicates that the columns are oriented nearly parallel to the glass tube. This anisotropic organization is attributed to the partial orientation of the gel fibers during the sample preparation or to directing effects of the glass tube during the LCST phase transition. This alignment was not studied in detail.

Preparation of the hydrogels and lyotropic liquid crystals. Hydrogels and lyotropic liquid crystal samples were prepared by weighing the PBI compounds in flasks and subsequent addition of the appropriate amount of water (20 wt% for lyotropic liquid crystals and 80 wt% for the hydrogels). The flasks were closed and stored at r.t. until the mixtures became homogeneous (ca. 24 h). In the case of the lyotropic samples, the mixtures were treated with a spatula to ensure the homogenous distribution of water in the PBI material.

The hydrogels based on mixtures of **PBI 1** and **PBI 2** were prepared as follows: **PBI 1** and **PBI 2** were mixed in the desired molar ratio by dissolving in dichloromethane. Subsequent removal of the solvent resulted in an amorphous mixed solid that was dried under vacuum. An appropriate amount of pure water (80 wt%**)** was added to the PBIs mixture in a flask that was closed and stored overnight at r.t..

2. Synthetic procedures

PBI 2 was synthesized according to our previously reported procedure.[S2] **PBI 1** was prepared according to Scheme S1. 2-bromo-1,3-diiodo-5-nitrobenzene (**1**)[S3] and 3-(2-(2-methoxyethoxy)ethoxy)prop-1-yne (**5**)[S4] were synthesized according to literature procedures.

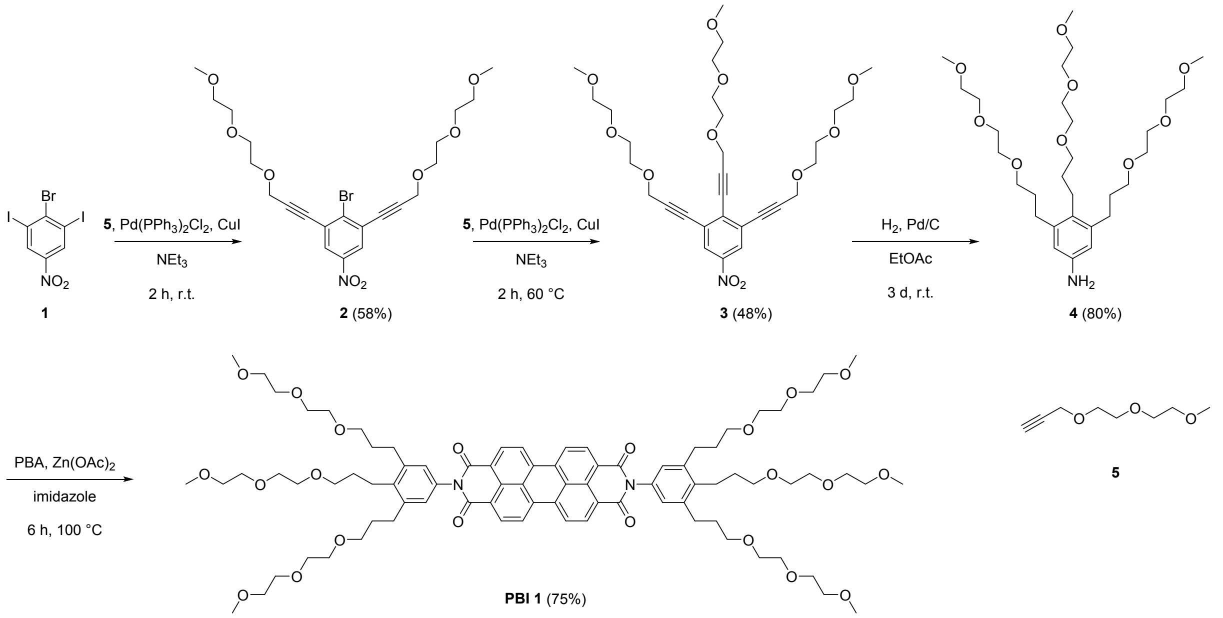

Scheme S1 Synthetic route for **PBI 1**. PBA: perylene-3,4:9,10-tetracarboxylic acid bisanhydride.

2-Bromo-1,3-bis(3-(2-(2-methoxyethoxy)ethoxy)prop-1-yn-1-yl)-5-nitrobenzene (**2**). 2-bromo-1,3-diiodo-5-nitrobenzene (**1**) (500 mg, 1.10 mmol), $Pd(PPh_3)_2Cl_2$ (39 mg, 55 μmol) and CuI (21 mg, 110 μmol) were dissolved in 10 mL of degassed triethylamine under argon atmosphere. Then, 3-(2-(2-methoxyethoxy)ethoxy)prop-1-yne (**5**) (435 mg, 2.75 mmol) was added dropwise upon stirring. The reaction mixture was allowed to stir for 2 hours at room temperature. Removal of the solvent and purification of the residue by column chromatography (ethyl acetate/n-hexane, 7:3) afforded **2** as a brownish oil (330 mg, 0.64 mmol, 58% yield).

^1H NMR (400 MHz, $CDCl_3$, δ): 8.21 (s, 2H, ArH), 4.50 (s, 4H, C*H*$_2$), 3.83-3.72 (m, 8H, CH_2), 3.69-3.56 (m, 8H, CH_2), 3.39 (s, 6H, CH_3); ^{13}C NMR (100 MHz, $CDCl_3$, δ): 146.5, 135.4, 127.5, 127.1, 93.4,

83.3, 72.1, 70.8, 70.7, 69.7, 59.3, 59.2; HRMS (ESI, m/z): $[M + Na]^+$ calcd for $C_{22}H_{28}BrNO_8Na$, 536.0891; found, 536.0880.

1,2,3-Tris(3-(2-(2-methoxyethoxy)ethoxy)prop-1-yn-1-yl)-5-nitrobenzene (**3**). Compound **2** (330 mg, 0.64 mmol), $Pd(PPh_3)_2Cl_2$ (23 mg, 32 μmol) and CuI (12 mg, 66 μmol) were dissolved in 15 mL of the degassed solvent mixture triethylamine /tetrahydrofuran (2:1) under argon atmosphere. After heating to 60 °C 3-(2-(2-methoxyethoxy)ethoxy)prop-1-yne (**5**) (132 mg, 0.83 mmol) was added dropwise upon stirring. Then, the reaction mixture was allowed to stir for 2 hours at 60 °C. Removal of the solvent and purification of the residue by column chromatography (dichloromethane/methanol, 97:3) afforded **3** as a brownish oil (180 mg, 0.30 mmol, 48% yield).

^1H NMR (400 MHz, $CDCl_3$, δ): 8.21 (s, 2H; ArH), 4.54 (s, 2H, CH_2), 4.48 (s, 4H, CH_2), 3.83–3.54 (m, 24H, CH_2), 3.38 (s, 6H, CH_3), 3.37 (s, 3H, CH_3)**;** ^{13}C NMR (100 MHz, $CDCl_3$, δ): 146.5, 133.3, 127.3, 126.4, 99.0, 92.4, 83.0, 82.9, 72.1, 70.8, 70.8, 70.6, 70.6, 69.6, 69.5, 59.3, 59.2, 59.2, 59.2; HRMS (ESI, m/z): $[M + Na]^+$ calcd for $C_{30}H_{41}NO_{11}Na$, 614.2572; found, 614.2559.

3,4,5-Tris(3-(2-(2-methoxyethoxy)ethoxy)propyl)aniline (**4**). Compound **3** (180 mg, 0.30 mmol) was dissolved in 20 mL of degassed ethyl acetate. 100 mg of Pd/C were added and the solution was placed into an autoclave under argon atmosphere before. The argon atmosphere was replaced by H_2 (10 bar) and the reaction mixture was allowed to stir for three days at room temperature. Then, the reaction mixture was filtered over celite and the solvent was removed by rotatory evaporation. The residue was purified by column chromatography (dichloromethane/methanol, 97:3) to afford **4** as brownish oil (137 mg, 0.24 mmol, 80% yield).

^1H NMR (400 MHz, $CDCl_3$, δ): 6.46 (s, 2H, ArH), 3.68–3.46 (m, 30H, CH_2), 3.38 (s, 6H, CH_3), 3.38 (s, 3H, CH_3), 2.57 (m, 6H, CH_2), 1.83 (m, 4H, CH_2), 1.70 (m, 2H, CH_2); ^{13}C NMR (100 MHz, $CDCl_3$, δ): 143.1, 141.4, 129.1, 115.1, 72.1, 71.4, 70.9, 70.8, 70.8, 70.7, 70.4, 70.3, 59.2, 59.2, 31.5, 31.2, 29.4, 24.5; HRMS (ESI, m/z): $[M + H]^+$ calcd for $C_{30}H_{56}NO_9$, 574.3950; found, 574.3952.

***N,N'*-[3,4,5-Tris(3-(2-(2-methoxyethoxy)ethoxy)propyl)phenyl]-perylene-3,4:9,10-tetracarboxylic acid bisanhydride** (**PBI 1**). Perylene-3,4:9,10-tetracarboxylic acid bisanhydride (77 mg, 0.20 mmol), compound **4** (250 mg, 0.44 mmol), $Zn(OAc)_2$ (143 mg, 0.79 mmol) and 3 g of imidazole were placed into a 10 mL round bottom flask and the mixture was stirred for 6 hours

at 100 °C under nitrogen atmosphere. Few drops of methanol were added to avoid imidazole crystallization and the reaction mixture was allowed to cool down to room temperature. The crude product was purified by column chromatography (dichloromethane/methanol, 97:3) to afford **PBI 1** as red waxy solid (231 mg, 0.15 mmol, 75% yield).

^{1}H NMR (400 MHz, $CDCl_3$, δ): 8.75 (d, *J* = 8 Hz, 4H, ArH), 8.70 (d, *J* = 8 Hz, 4H, ArH), 7.01 (s, 4H, ArH), 3.72–3.50 (m, 60H, CH_2), 3.40 (s, 6H, CH_3), 3.34 (s, 12H, CH_3), 2.77 (m, 12H, CH_2), 1.93 (m, 8H, CH_2), 1.84 (m, 4H, CH_2); ^{13}C NMR (100 MHz, $CDCl_3$, δ): 163.8, 141.7, 139.0, 135.1, 132.7, 132.0, 130.0, 126.9, 126.9, 123.8, 123.5, 72.2, 72.1, 71.4, 70.9, 70.8, 70.8, 70.7, 70.5, 70.3, 59.3, 59.2, 31.0, 30.8, 29.4, 25.3; HRMS (ESI, m/z): $[M + H]^+$ calcd for $C_{84}H_{115}N_2O_{22}$, 1503.7936; found, 1503.7933. Elemental analysis calcd. for $C_{84}H_{114}N_2O_{22}$: C 67.09, H 7.64, N 1.86; found: C 66.73, H 7.62, N 1.81.

3. Supplementary Figures

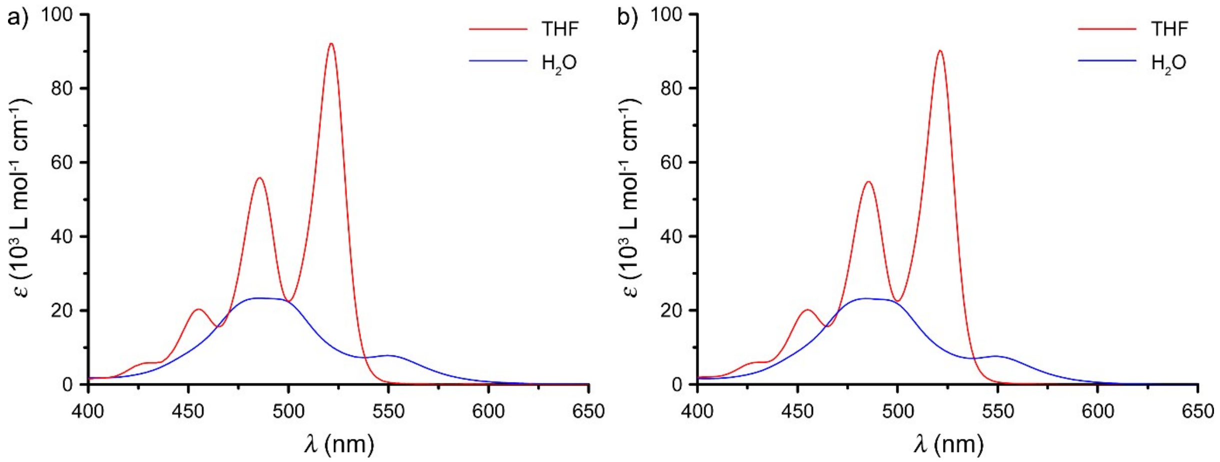

Fig. S1 UV/Vis absorption spectra of (a) **PBI 1** and (b) **PBI 2** in tetrahydrofuran (red) and water (blue). Concentration ~ 2-3 x 10^{-6} M.

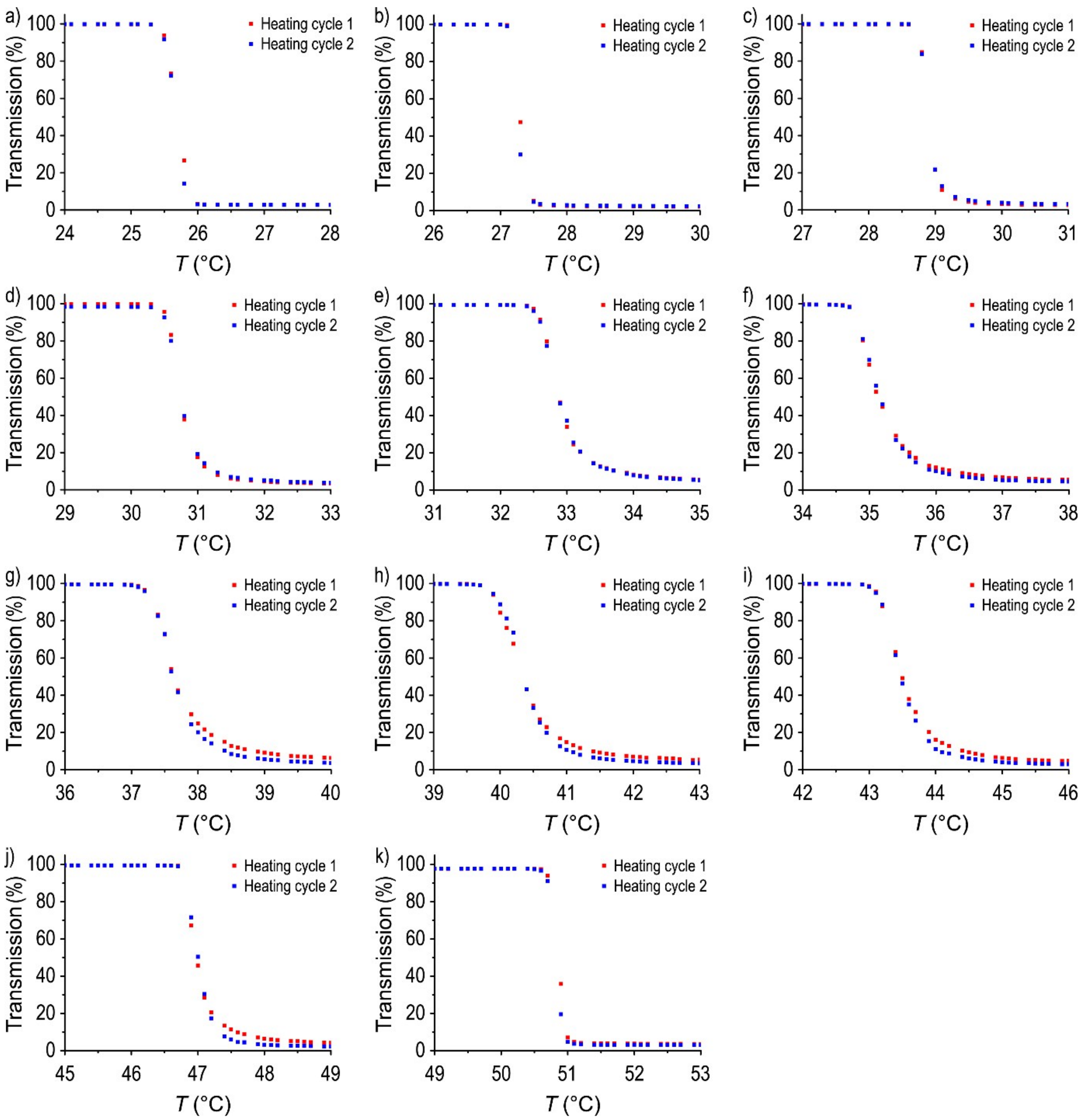

Fig. S2 Transmission at 800 nm as a function of the temperature of aqueous solutions of **PBI 1/PBI 2** (2.5 x 10^{-4} M) with the **PBI 1** molar fraction (χ) of (a) 0, (b) 0.1, (c) 0.2, (d) 0.3, (e) 0.4, (f) 0.5, (g) 0.6, (h) 0.7, (i) 0.8, (j) 0.9 and (k) 1. First and second heating are indicated in red and blue dots, respectively. Heating rate: 0.1 °C min^{-1}.

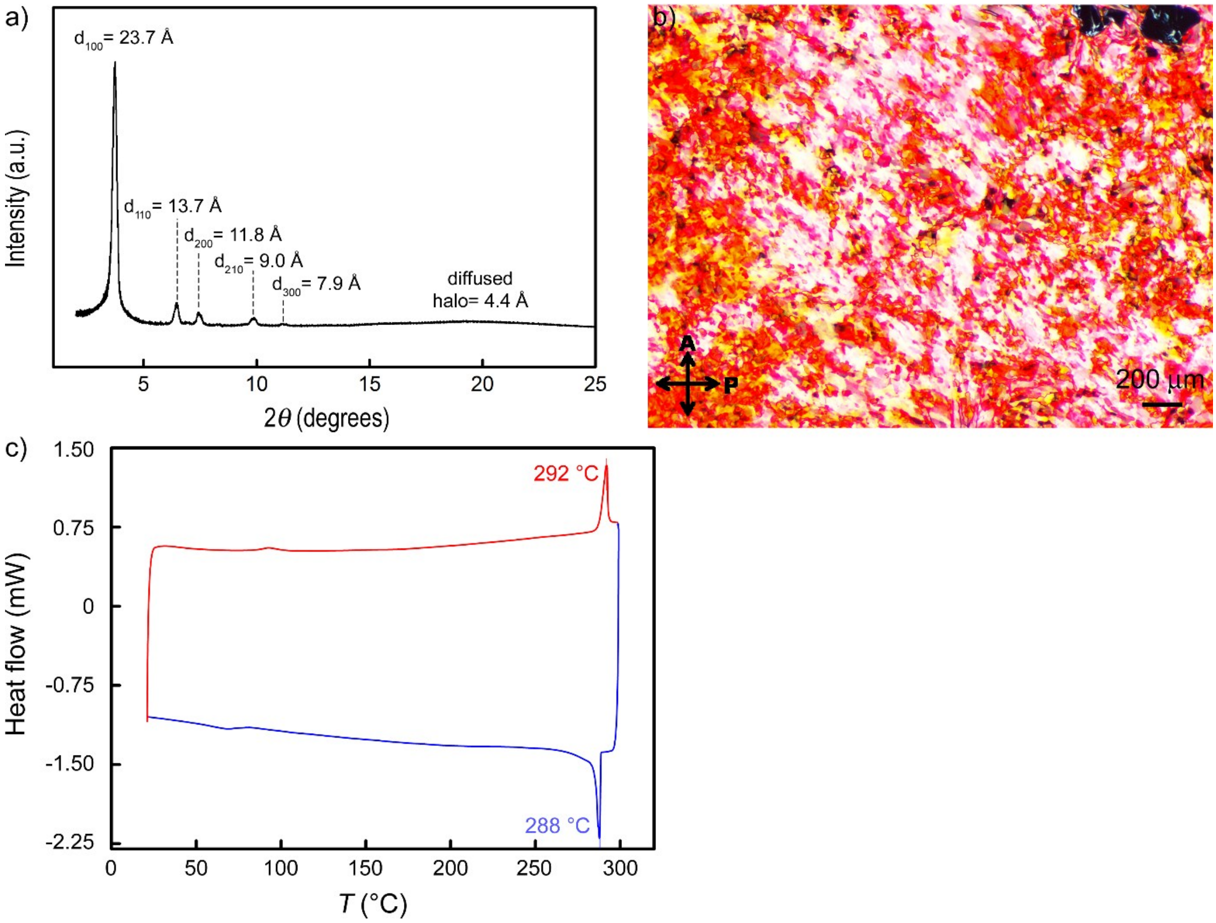

Fig. S3 (a) 1D plot of the XRD pattern of **PBI 1** at 150 °C. (b) POM image of **PBI 1** at 200 °C after cooling from the isotropic melt. (c) DSC thermogram of **PBI 1**. First cooling (blue) and second heating (red). The heating/cooling rate was 10 °C/min. Diffraction peaks were assigned according to the packing characteristics of a liquid-crystalline columnar hexagonal phase (a = 27.3 Å).

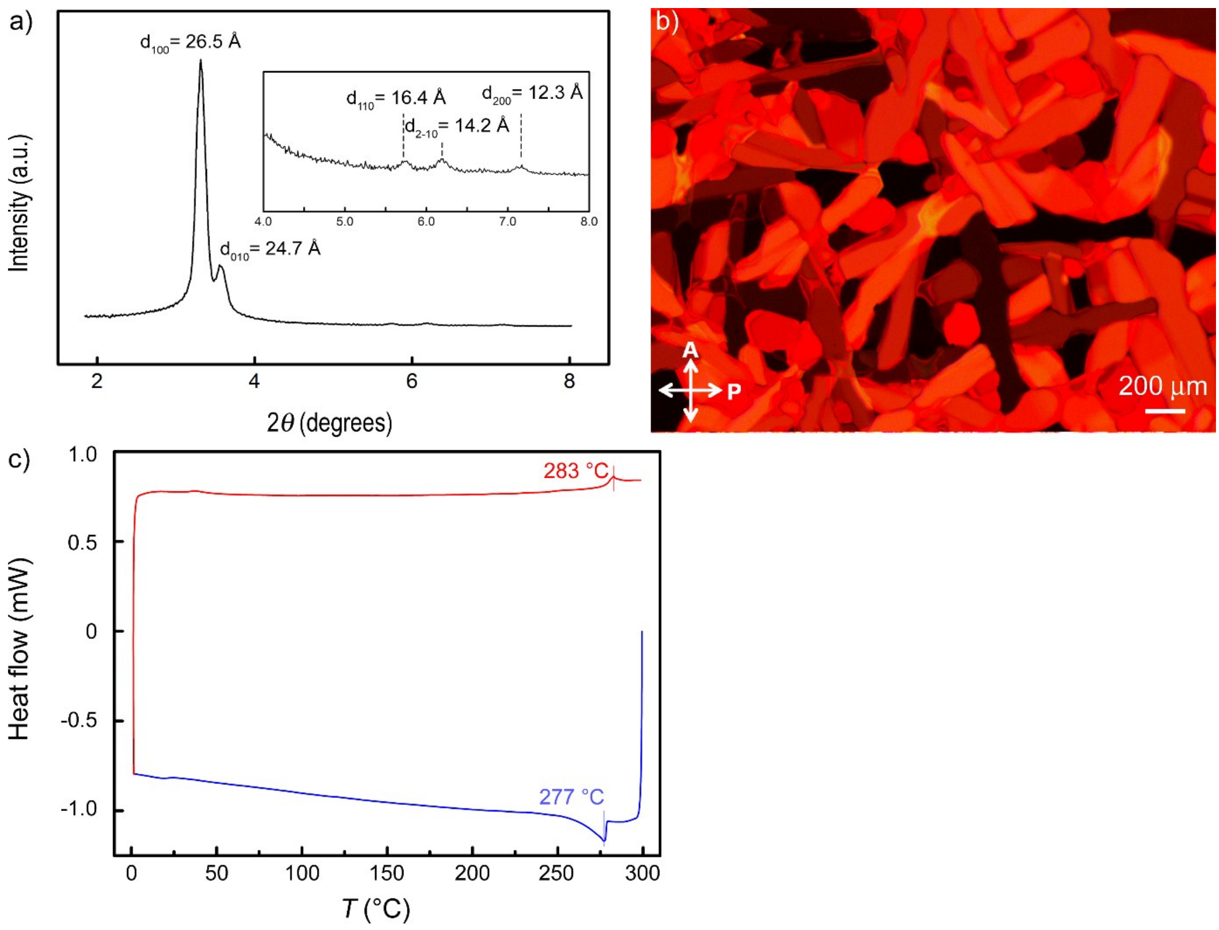

Fig. S4 (a) 1D plot of the XRD pattern of **PBI 2** at 100 °C**.** (b) POM image of **PBI 2** at 250 °C after cooling from the isotropic melt. (c) DSC thermogram of **PBI 2**. First cooling (blue) and second heating (red). The heating/cooling rate was 10 °C/min. The diffraction pattern for PBI 2 at 100 °C does not match with the common hexagonal, tetragonal and rectangular columnar phases, but shows consistence with the packing characteristics of a liquid-crystalline columnar oblique phase.

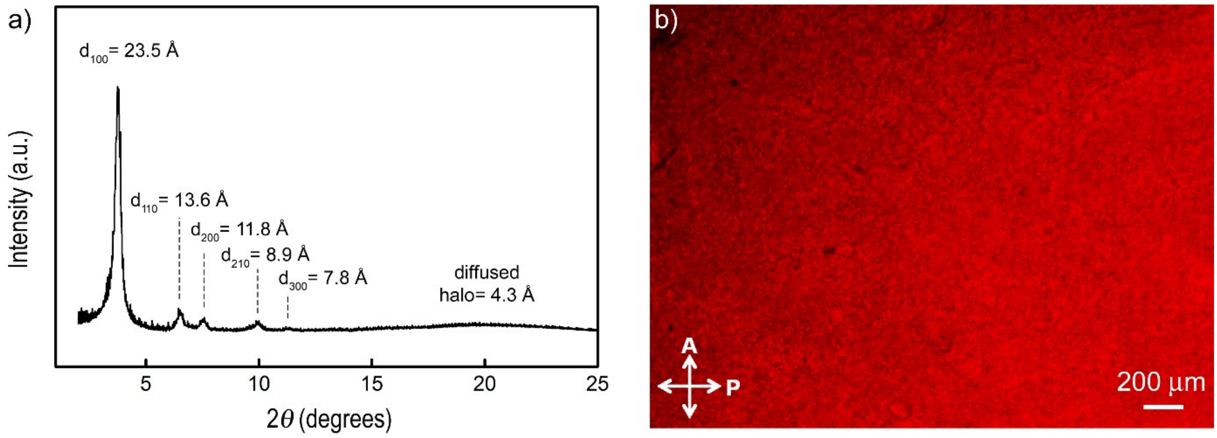

Fig. S5 (a) 1D plot of the XRD pattern of **PBI 1** with 20 w.t.-% of water at 70 °C. (b) POM image of **PBI 1** with 20 w.t.-% of water at 70 °C. Diffraction peaks were assigned according to the packing characteristics of a columnar hexagonal phase (a = 27.2 Å).

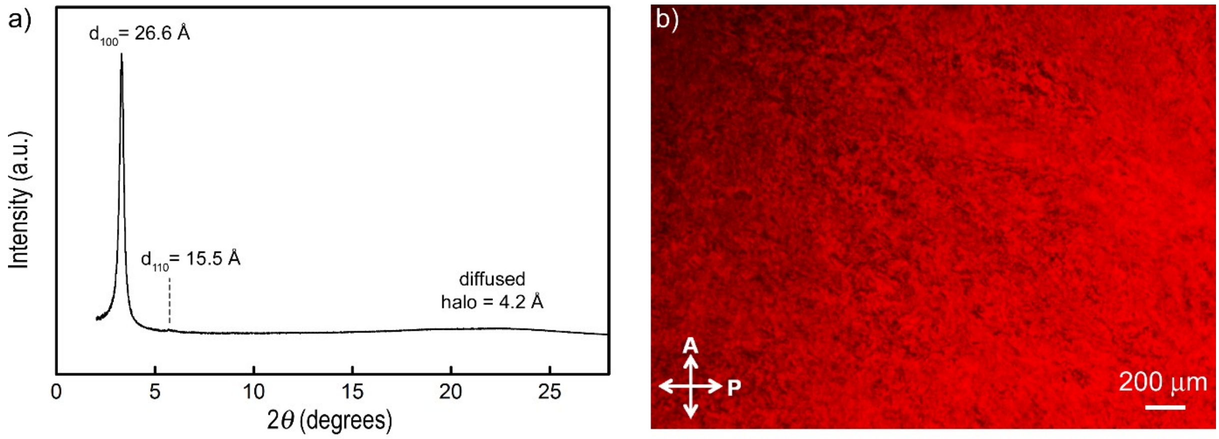

Fig. S6 (a) 1D plot of the XRD pattern of **PBI 2** with 20 w.t.-% of water at 50 °C. (b) POM image of **PBI 2** with 20 w.t.% of water at 40 °C. Diffraction peaks were assigned according to the packing characteristics of a liquid-crystalline columnar hexagonal phase (a = 30.7 Å).

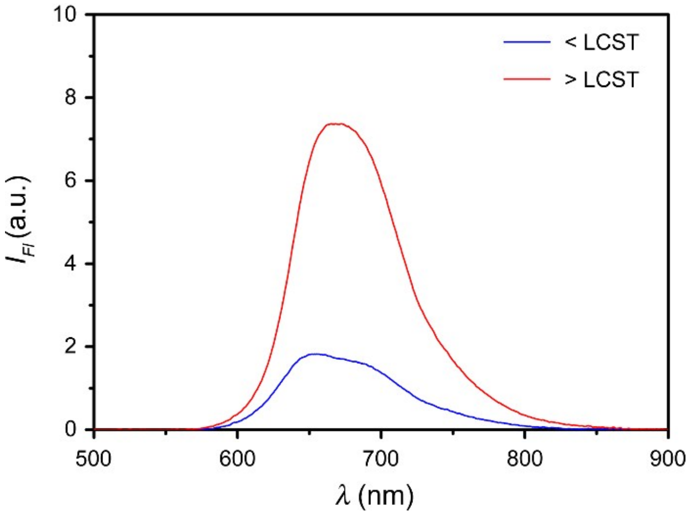

Fig. S7. a) Fluorescence spectra of **PBI 2** in water (80 w.t.-%) before (21 °C, blue) and after (35 °C, red) LCST phase transition. λ_{ex} = 380-420 nm.

The fluorescence spectra of the gels were recorded for film samples. The sample was sandwiched between two glass plates and the fluorescence was measured at different temperatures by a Fluorescence Microscope equipped with a hot stage. These films are too thick for the measurement of absorption.

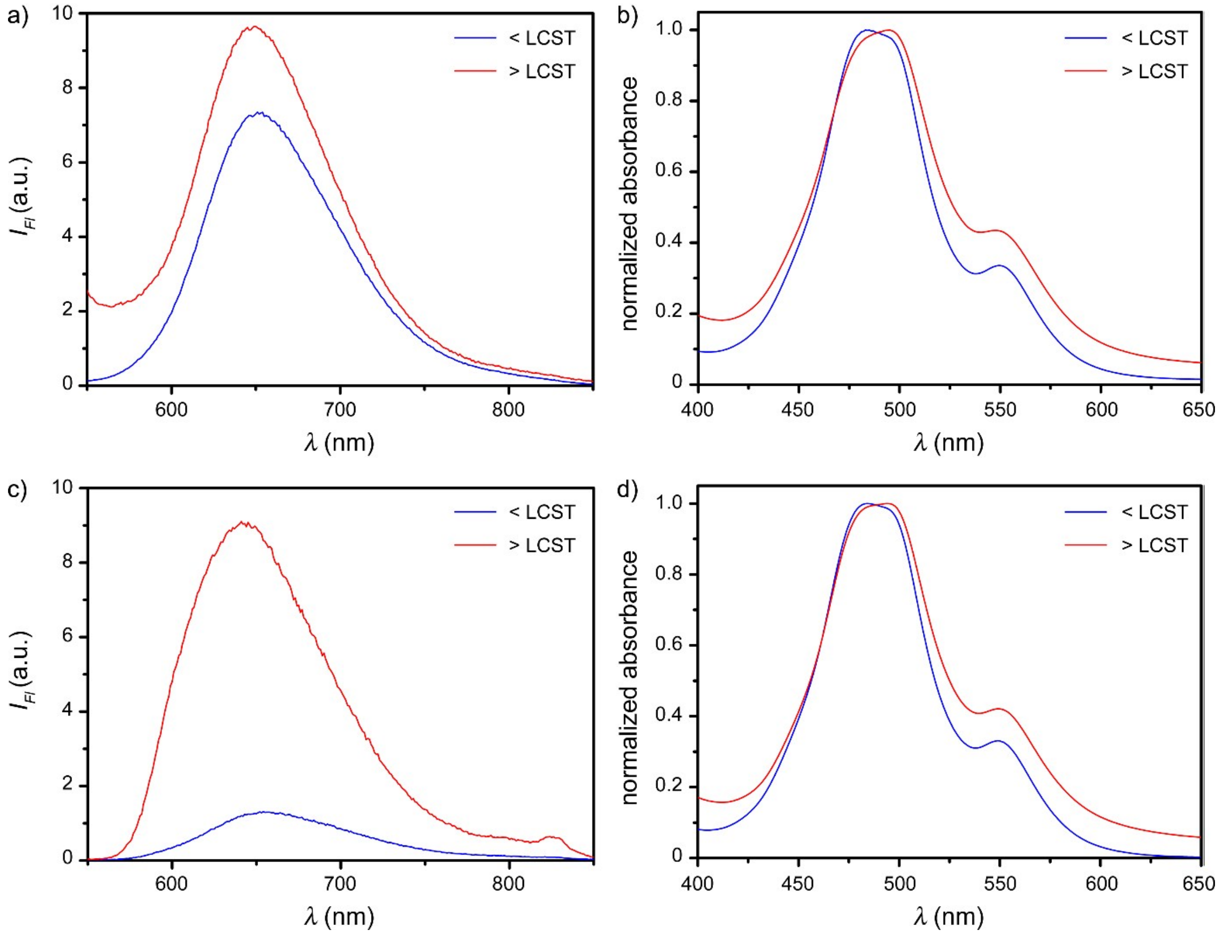

Fig. S8 a) Fluorescence spectra of **PBI 1** in water (8.2 x 10^{-5} M) at 40 (blue) and 55 °C (red). Excitation wavelength (λ_{ex} = 470 nm). b) UV/Vis absorption spectra of **PBI 1** in water (8.2 x 10^{-5} M) at 25 (blue) and 60 °C (red). c) Fluorescence spectra of **PBI 2** in water (8 x 10^{-5} M) at 25 (blue) and 35 °C (red). Excitation wavelength (λ_{ex} = 470 nm). d) UV/Vis absorption spectra of **PBI 2** in water (8 x 10^{-5} M) at 25 (blue) and 26 °C (red)

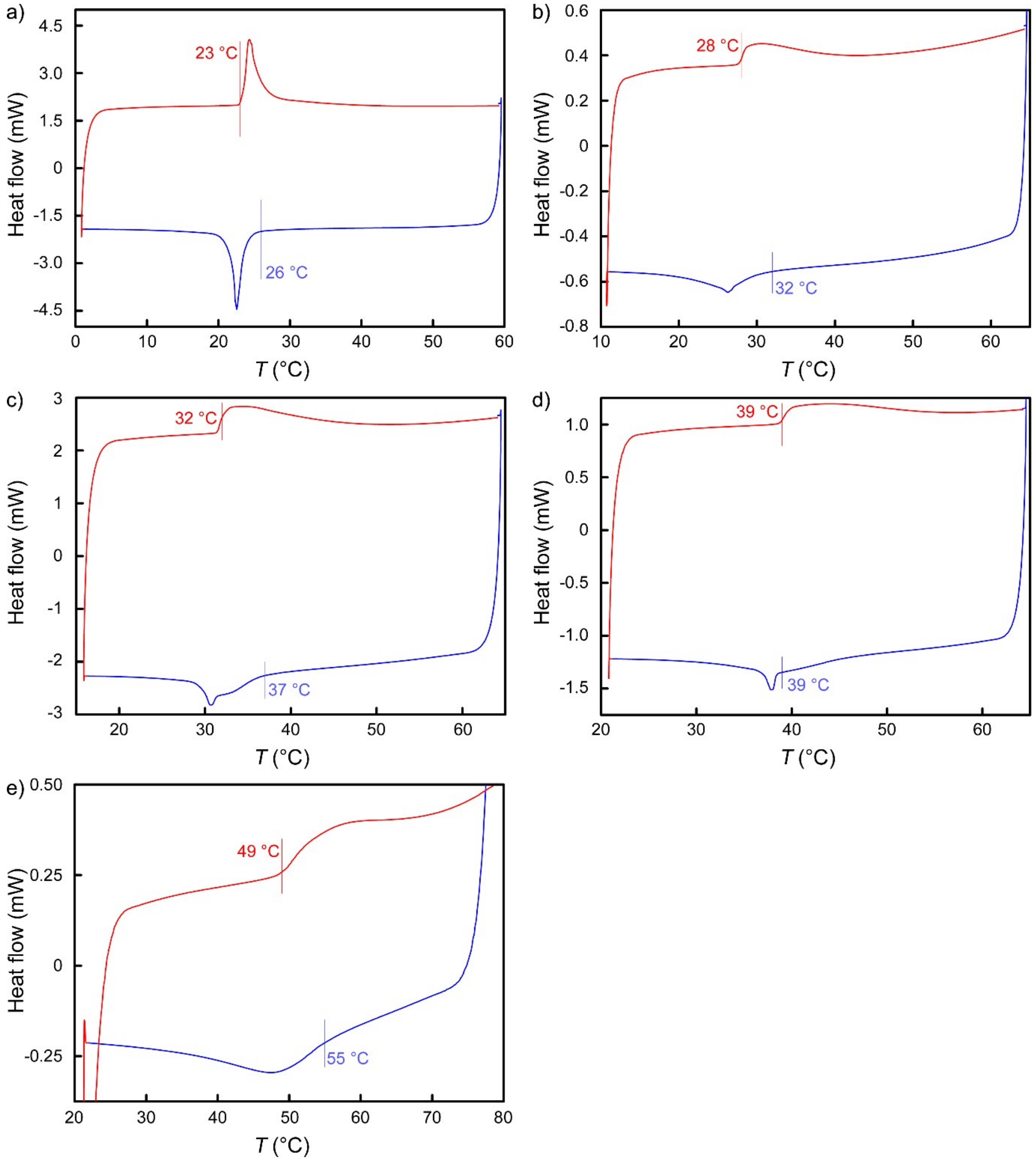

Fig. S9 DSC thermogramms of pure and mixed PBI hydrogels (80 w.t.-% water content) composed of (a) 100 % **PBI 2** and (b) 25 % **PBI 1** and 75 % **PBI 2**, (c) 50 % **PBI 1** and 50 % **PBI 2**, (d) 75 % **PBI 1** and 25 % **PBI 2** and (e) 100 % **PBI 1**. The second heating and the first cooling processes are indicated in red and blue, respectively. The heating and cooling rates were 5 °C/min.

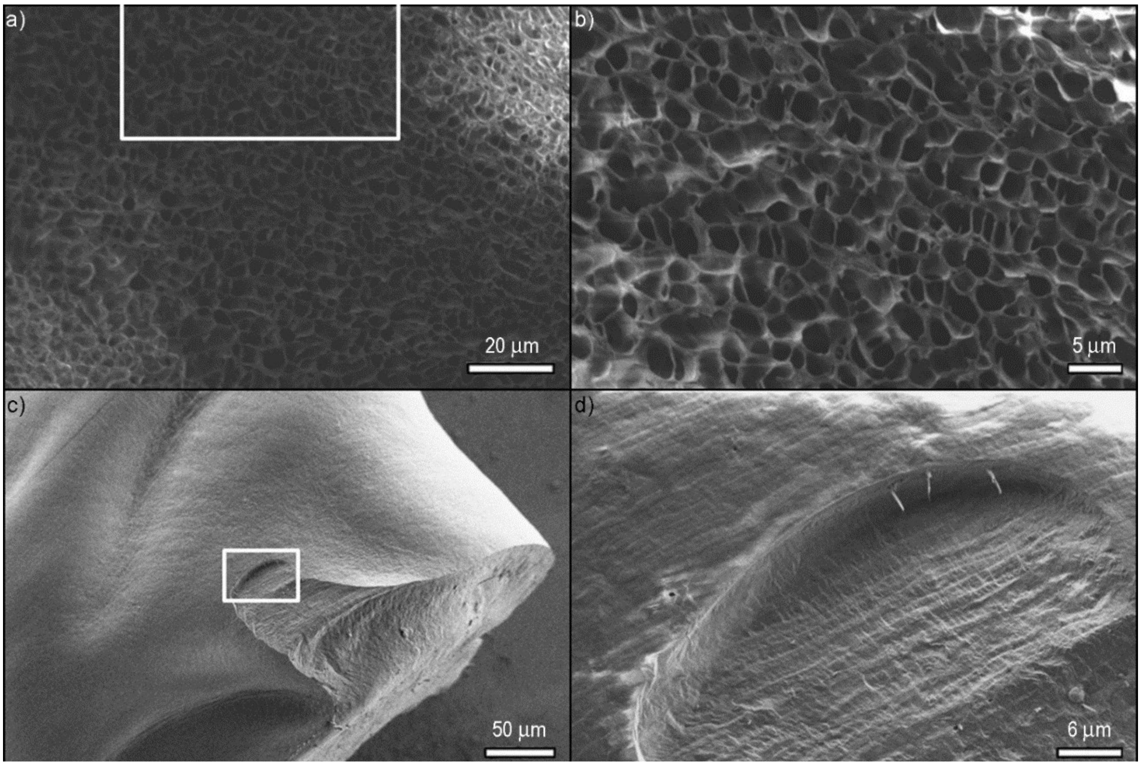

Fig. S10 Cryo-SEM-images of **PBI 1** in H_2O [c = 20 % (m/m)], (a,b) prepared at ambient conditions below the LCST and (c,d) above LCST, prepared at 60 °C. The white rectangles in (a) and (c) indicate the magnified regions shown in (b) and (d), respectively.

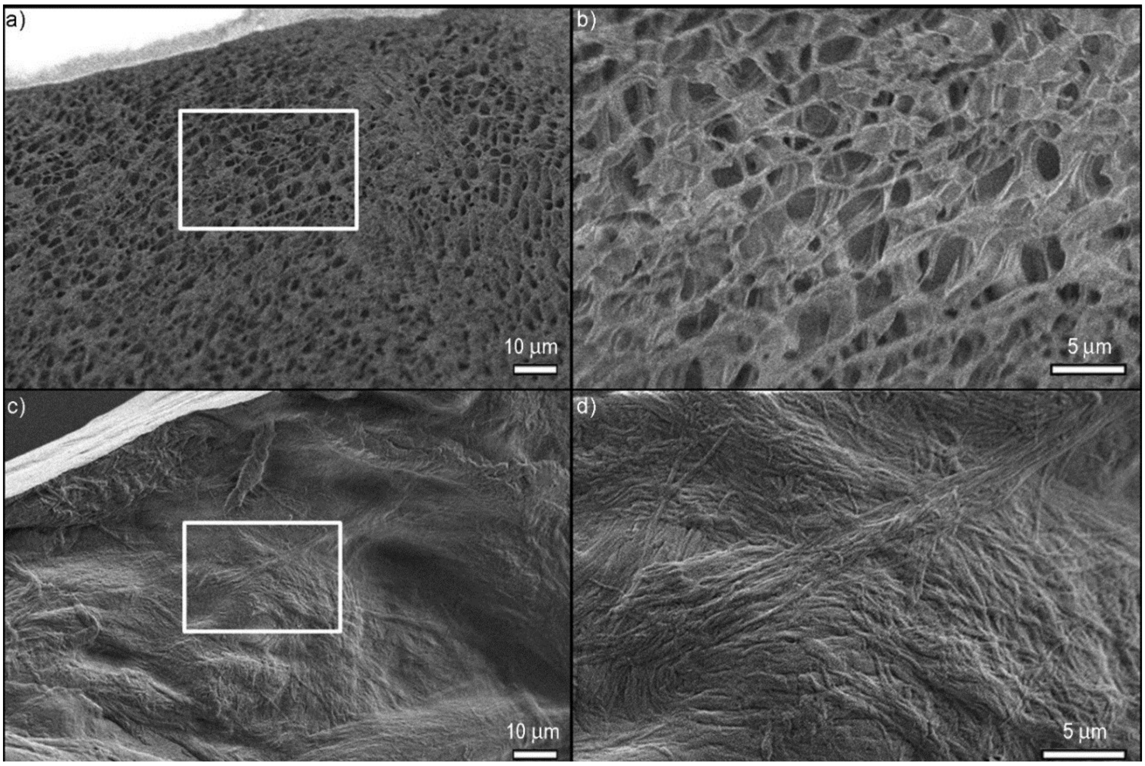

Fig. S11 Cryo-SEM-images of **PBI 2** in H_2O [c = 20 % (m/m)], (a,b) prepared at ambient conditions below the LCST and (c,d) above LCST, prepared at 30 °C. The white rectangles in (a) and (c) indicate the magnified regions shown in (b) and (d), respectively.

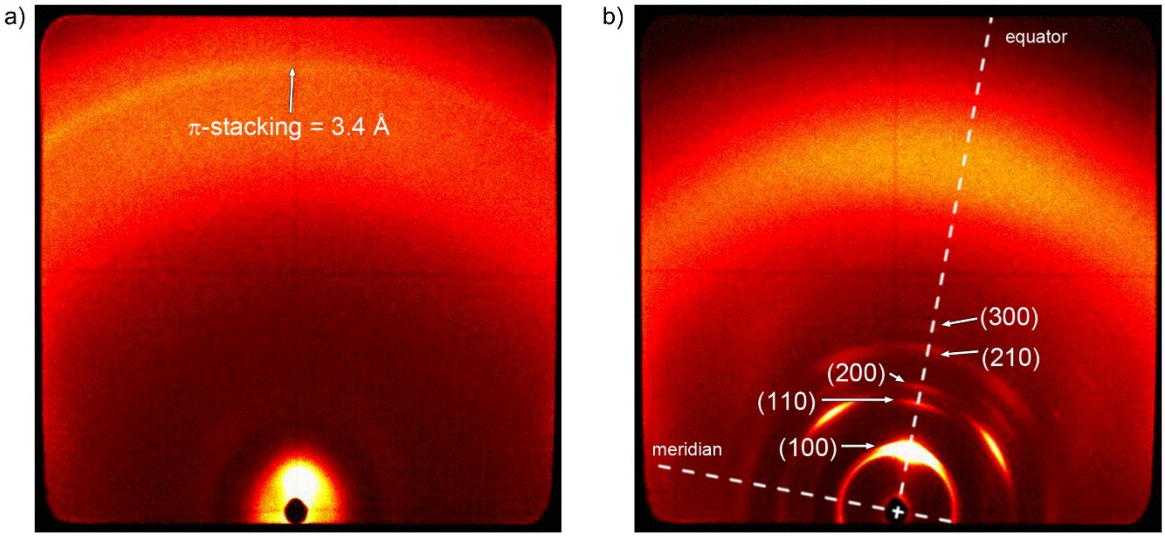

Fig. S12 Wide angle 2D X-ray diffraction patterns of **PBI 2** hydrogel (80 wt% water content) (a) below LCST, recorded at 20 °C, and (b) above LCST, recorded at 50 °C.

The X-ray diffraction patterns of **PBI 1** (Figure 4d in the manuscript) and **PBI 2** (Figure S10b) hydrogels above the LCST were assigned to columnar hexagonal phases. The columnar lattices and lengths of the respective unit cells are given in Table S1.

Table S1 Distances of the packing lattices of the **PBI 1** (at 70 °C) and **PBI 2** (at 50 °C) hydrogels obtained by XRD measurements after LCST phase transition.

Distances [Å]	(100)	(110)	(200)	(210)	(300)	*a*
**PBI 1**	26	15	13	9.8	-	30
**PBI 2**	25	14	12	9.3	8.2	29

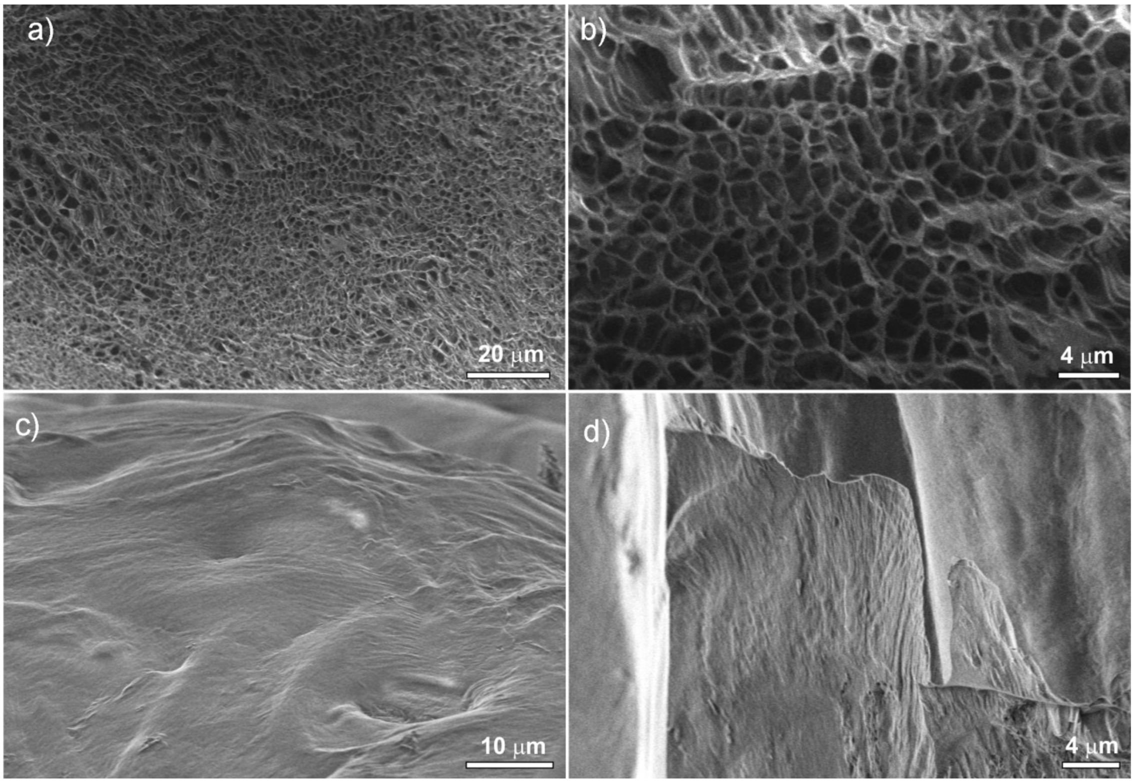

Fig. S13 Cryo-SEM-images of **PBI 1/PBI 2** (1:1) in H_2O [c = 20 % (m/m)], (a,b) prepared at ambient conditions below the LCST and (c,d) above LCST, prepared at 60 °C.

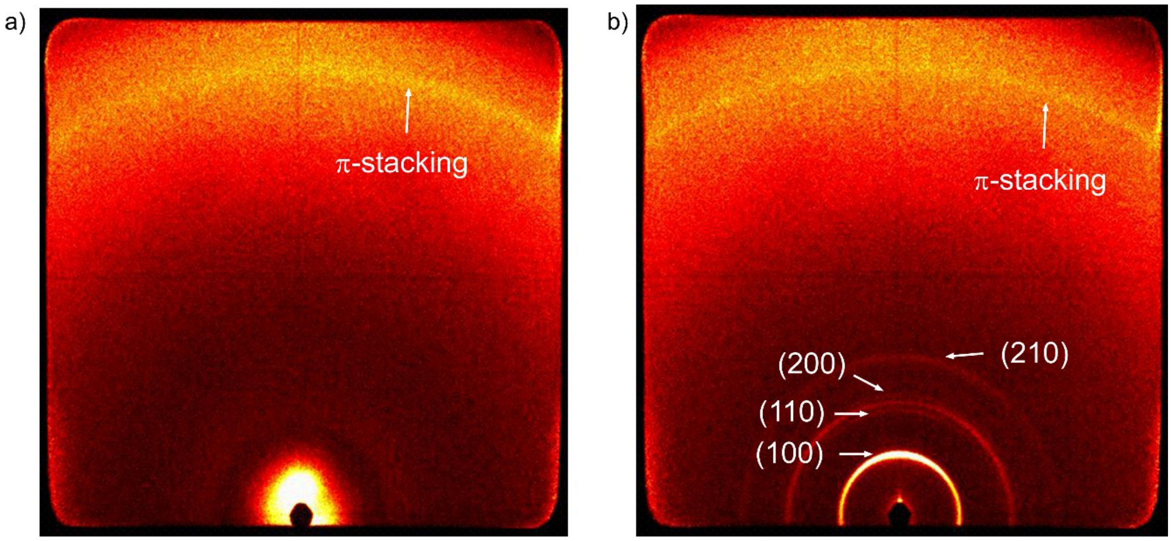

Fig. S14 Wide angle 2D X-ray diffraction patterns of **PBI 1/PBI 2** (1:1) hydrogel (80 wt% water content) (a) below LCST, recorded at 25 °C, and (b) above LCST, recorded at 50 °C.

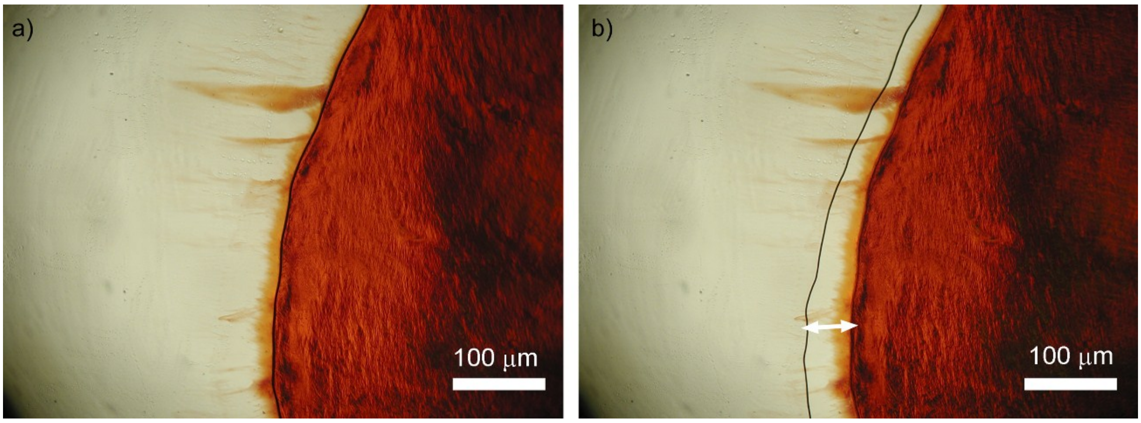

Fig. S15 Optical microscope images of **PBI 1/PBI 2** mixture (1:1) in H_2O (80 mw% water content) (a) below LCST (30 °C) and b) above LCST (45 °C). The release of water at the periphery of the gel is indicated with a white arrow.

4. Supplementary References

S1 R. Gvishi, R. Reisfeld and Z. Burshtein, *Chem. Phys. Lett*. 1993, **213**, 338.

S2 X. Zhang, S. Rehm, M. Safont-Sempere and F. Würthner, *Nat. Chem.* 2009, **1**, 623.

S3 W. Tao, S. Nesbitt and R. F. Heck, *J. Org. Chem.* 1990, **55**, 63.

S4 B. A. Scates, B. L. Lashbrook, B. C. Chastain, K. Tominaga, B. T. Elliott, N. J. Theising, T. A. Baker and R. W. Fitch, *Bioorg. Med. Chem.* 2008, **16**, 10295.
